# Metabolomic Insights into Energy Utilization Strategies of Asiatic Toads (*Bufo gargarizans*) During Hibernation

**DOI:** 10.3390/ani15030403

**Published:** 2025-01-31

**Authors:** Hui Ma, Chengzhi Yan, Zhiping Mi

**Affiliations:** Key Laboratory of Southwest China Wildlife Resources Conservation (Ministry of Education), China West Normal University, Nanchong 637009, China; mahui102@cwnu.edu.cn

**Keywords:** hibernation, metabolomics, *Bufo gargarizans*, TCA cycle, energy conservation

## Abstract

**Simple Summary:**

Hibernation is a critical survival strategy that enables amphibians to endure extreme environmental conditions by dramatically reducing their metabolic rate. In this study, we investigated the metabolic changes in Asiatic toads (*Bufo gargarizans*) during hibernation. Through comprehensive analysis of their blood chemistry, we found that most metabolic processes were significantly reduced, indicating an overall decrease in activity. Notably, while pathways involving amino acids and carbohydrates were downregulated, lipid metabolism demonstrated a unique response. The increased β-oxidation of fatty acids—such as palmitoleic acid, arachidonic acid, and sodium caprylate—indicates a metabolic shift towards lipid-based energy utilization. This adjustment enables the toads to sustain themselves without food intake for prolonged periods. This research contributes to a broader understanding of how amphibians efficiently manage energy resources under stress, highlighting the intricate balance of the biological processes that support life in challenging environments.

**Abstract:**

Hibernation is a crucial adaptive strategy for amphibians, facilitating survival in harsh environmental conditions by lowering metabolic rates and reducing energy use. This study employed GC-MS and LC-MS metabolomics to systematically analyze the serum metabolome of *Bufo gargarizans* during hibernation, aiming to uncover its metabolic adaptation mechanisms. A total of 136 differentially expressed metabolites (DEMs) were identified, of which 115 were downregulated and 21 upregulated, mainly involved in amino acid, carbohydrate, and lipid metabolism. KEGG pathway analysis showed that most metabolic pathways were inhibited in the hibernating group, underscoring a significant reduction in overall metabolic activity. Notably, while amino acid and carbohydrate metabolism were significantly reduced, lipid metabolism exhibited a distinctive adaptive response. Enhanced β-oxidation of fatty acids, including palmitoleic acid, arachidonic acid, and sodium caprylate, suggests a metabolic shift toward lipid-based energy utilization. The reduction in key metabolites like fumaric acid and succinic acid in the TCA cycle further supports the hypothesis of reduced energy requirements. These results enhance our current understanding of amphibian hibernation metabolisms and provide a targeted approach for future mechanistic investigations.

## 1. Introduction

Hibernation is a critical adaptive strategy in response to the challenges posed by low environmental temperatures and a scarcity of food and water [1]. This state is characterized by significantly reduced body temperature, heart rate, respiration, and metabolic rate [2]. Hibernation is observed across diverse taxa, including mammals, birds, and various amphibians and reptiles. By effective energy conservation, hibernation enables animals to maintain essential physiological functions, such as basic metabolic processes, cardiac function, neural activity, and cellular homeostasis [3,4]. Investigating the phenomenon of animal hibernation not only advances our understanding of adaptation to extreme environments, but also provides critical insights into species conservation and ecological management strategies.

As ectotherms, amphibians employ hibernation as a vital strategy to evade extreme cold, dryness, and predators during winter. Studies have elucidated significant physiological alterations during this period, encompassing immune and digestive functions, energy metabolism, and antioxidant defense [5,6]. In *Bufo gargarizans*, core clock genes in the liver and corticosterone levels in the plasma maintain persistent circadian rhythms during hibernation, unlike the halted rhythms in the brain. This suggests a synergistic role of the hepatic and adrenal clocks in regulating metabolic demands for survival [7]. Similarly, in hibernating *Nanorana parkeri*, the myocardium and skeletal muscles show distinct gene expression profiles related to stress response, defense mechanisms, and muscle contraction, alongside differences in metabolites associated with amino acid metabolism when compared to their active summer counterparts [8]. Additionally, some hibernating amphibians exhibit increased oxidative stress, marked by elevated lipid peroxidation and protein carbonylation, coupled with enhanced antioxidant defenses [9,10]. Overall, these findings indicate that amphibians adapt to challenging winter environments through coordinated physiological processes throughout hibernation.

Although the physiological adaptations related to amphibian hibernation have been extensively documented, a comprehensive understanding of the underlying metabolic processes remains essential. During hibernation, amphibians stop feeding and rely on significantly reduced metabolic rates alongside the mobilization of energy reserves, primarily glycogen and lipids [11,12]. The ability of amphibians to endure several months of hibernation largely depends on these lipid reserves, with glycogen stores providing additional survival time [13,14]. For instance, in *Rana temporaria*, lipids from liver and fat bodies demonstrated utilization rates of 93% and 69.6%, respectively, contributing to 80.4% of the energy expended during hibernation. Concurrently, more than half of liver glycogen reserves were depleted during this period [15]. Similar utilization patterns have been documented in other related amphibian species, underscoring a potentially conserved metabolic strategy among hibernating amphibians [16,17]. With the advancement of nuclear magnetic resonance (NMR) and mass spectrometry (MS) technologies, metabolomics has provided a powerful tool for the comprehensive analysis of small molecules, known as metabolites [18]. This approach has been utilized to detect metabolic changes in tissues such as muscle, liver, and colon contents during hibernation [8,19]. Nevertheless, while some metabolic alterations have been identified, a deeper understanding of the specific metabolic pathways, the dynamic changes in metabolite levels, and their broader biochemical significance at the organismal level during hibernation remains necessary.

In this study, we investigate the metabolic distinctions between hibernating and active Chinese toads (*Bufo gargarizans*) at the whole-body level. This species is widely distributed across China and is considered an ideal model for studying physiology, behavior, and evolutionary biology [20,21]. We hypothesize that hibernation induces significant alterations in metabolic pathways, such as shifts toward energy-efficient processes and the suppression of non-essential activities, which facilitate the toads’ survival in extreme environments. By employing dual-platform metabolomics approaches, specifically gas chromatography–mass spectrometry (GC-MS) and liquid chromatography–mass spectrometry (LC-MS), we conduct a detailed analysis of metabolites and their relative abundances in whole-body serum samples of toads, facilitating a more comprehensive and precise evaluation [22]. Through this investigation, we aim to unravel the specific mechanisms of metabolic regulation during hibernation, thus offering a novel understanding of amphibian ecological adaptation strategies and providing valuable insights into broader biological responses.

## 2. Materials and Methods

### 2.1. Sample Preparation

Twelve sexually mature male *B. gargarizans* were collected from agricultural fields in Nanchong City, China, on the evening of 18 October 2023. The toads were randomly assigned to two groups, each consisting of six individuals: a non-hibernation group and a hibernation group. Prior to the experiment, all toads were acclimated at 23 °C for three days. For the non-hibernation group, toads were euthanized via double pithing, following the guidelines of Institutional Animal Care and Use Committee of China West Normal University (approval code: CWNU2021D006). Next, 1 mL blood samples were obtained via cardiac puncture. The blood was left at room temperature for 1 h and then centrifuged at 3000 rpm and 4 °C for 10 min to isolate the serum, which was subsequently stored at −80 °C for future analysis. The hibernation group was housed in a container (37 cm × 35 cm × 15 cm) filled with moist soil to simulate natural hibernation conditions. The container was placed in an incubator where the temperature was gradually decreased from 23 °C to 4 °C over a seven-day period, allowing the toads to acclimate to the lower temperature. The toads remained at 4 °C for 60 days [19]. Serum was then collected using the same method as the non-hibernation group.

### 2.2. Metabolite Extraction

Serum samples stored at −80 °C were thawed in an ice–water mixture to minimize degradation. For each sample, 150 μL of serum was mixed with 600 μL of a precooled precipitant solution (methanol/acetonitrile, 2:1) containing internal standards at 4 μg/mL. After vortexing for 1 min, the mixture was subjected to ultrasonication in an ice–water bath for 10 min to extract the metabolites, followed by precipitation at −40 °C for 2 h. The samples were then centrifuged at 13,000 rpm and 4 °C for 20 min [23]. The clear supernatant (150 μL) was filtered into LC vials and stored at −80 °C until LC-MS analysis. Simultaneously, an equal volume of the supernatant was concentrated by centrifugal evaporation and then derivatized with methoxylamine hydrochloride in pyridine at 37 °C for 1 h. This was followed by a second derivatization step using BSTFA and n-hexane, along with internal standards, at 70 °C for another hour. After allowing the samples to reach room temperature for 30 min, they were ready for GC-MS analysis. All reagents were pre-cooled to −20 °C to ensure consistency throughout the process. The quality control (QC) samples, made by combining extracts from all individual samples, were vortexed thoroughly to ensure homogeneity. For system stability and data reproducibility assessment, 3 μL of the QC sample was injected at regular intervals (every six experimental samples) during LC-MS analysis, while 1 μL was injected during GC-MS analysis.

### 2.3. GC-MS and LC-MS Analysis

The analytical platform for this experiment included a Waters ACQUITY UPLC I-Class Plus (Milford, MA, USA)/Thermo QE HF system (Waltham, MA, USA) and an Agilent 7890B-5977A system (Santa Clara, CA, USA). The GC-MS parameters, including both programmed temperature and mass spectrometry settings, were applied as described in our previous study [21]. For LC-MS, separation was performed using an ACQUITY UPLC HSS T3 column (1.8 μm, 2.1 × 100 mm) in both positive and negative ion modes. The gradient elution used solvents A (water with 0.1% formic acid) and B (acetonitrile with 0.1% formic acid), with the following profile: 0.01 min at 5% B, kept until 2 min; increased to 30% B at 4 min, 50% B at 8 min, 80% B at 10 min, and 100% B at 14 min, held until 15 min, then returned to 5% B by 15.1 min, finishing at 16 min. The flow rate was 0.35 mL/min with the column temperature at 45 °C. The samples were analyzed at 10 °C with an injection volume of 3 μL. The mass spectrometry settings used were based on the methods detailed by Wu et al. [24].

### 2.4. Data Processing and Analysis

Raw GC-MS data were converted from .D format to .abf format to facilitate rapid data retrieval. Peak detection, identification, and alignment were performed using MS-DIAL software (DIAL version 4.24). Metabolite characterization was conducted with the LUG database, resulting in a three-dimensional data matrix that included sample information, peak names, retention times, retention indices, and signal intensities. Differentially expressed metabolites (DEMs) were identified through 7-fold cross-validation and 200-response permutation tests. For LC-MS data, baseline filtering, retention time correction, and normalization were executed using Progenesis QI v2.3 software. Compound identification utilized the HMDB database, and qualitatively inaccurate data were excluded. Data matrices from both the GC-MS and LC-MS platforms were subjected to principal component analysis (PCA) using R software (R version 4.2.2) in an RStudio environment to evaluate the overall distribution. Orthogonal partial least squares discriminant analysis (OPLS-DA) was subsequently applied using the ropls package in R to identify DEMs, with significant metabolites selected based on VIP > 1 and *p* < 0.05. DEMs were further analyzed for Kyoto encyclopedia of genes and genomes (KEGG) pathway enrichment following the methodology of Wu et al. [24].

## 3. Results

### 3.1. Characteristics of Hibernation and Non-Hibernation Groups

**Figure 1:**
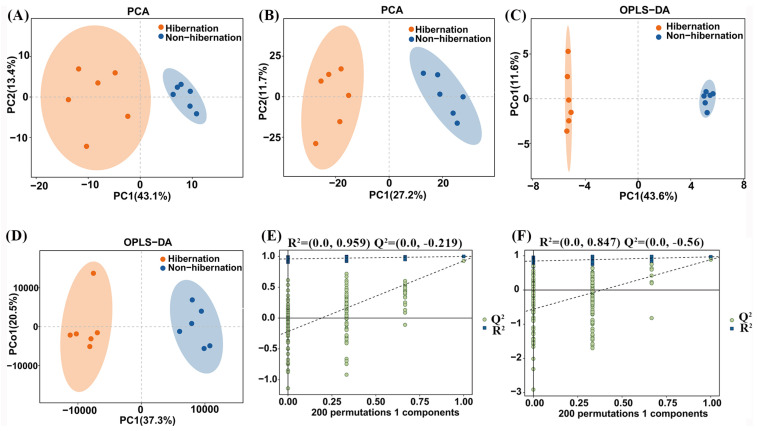
Score plots for *B. gargarizans* comparing hibernation and non-hibernation groups: principal component analysis (PCA) based on GC-MS (**A**) and LC-MS (**B**); orthogonal partial least squares discriminant analysis (OPLS-DA) based on GC-MS (**C**) and LC-MS (**D**); response permutation tests based on GC-MS (**E**) and LC-MS (**F**).

To elucidate the metabolic differences in *B. gargarizans* during hibernation and non-hibernation periods, we performed GC-MS and LC-MS metabolomics on the blood samples. The resulting dataset was systematically analyzed using PCA and OPLS-DA. The resulting dataset, containing normalized metabolite features obtained from both GC-MS and LC-MS analyses, was systematically analyzed using PCA to assess overall sample distribution and OPLS-DA to identify class-specific differences. The PCA effectively distinguished the metabolic profiles of hibernation versus non-hibernation groups in both GC-MS and LC-MS analyses, indicating significant metabolic shifts (Figure 1A,B). This separation was further validated by the OPLS-DA analysis (Figure 1C,D). In the OPLS-DA plots, we evaluated the model’s performance using the permutation test, examining metrics such as R^2^Y (cum), Q^2^ (cum), and Q^2^ to evaluate the model’s interpretive and predictive power as well as its robustness. Also, R^2^ values were employed to assess the model’s goodness of fit. For GC-MS analysis, the R^2^Y value was 0.999, and the Q^2^ value was 0.927 (Table S1). In contrast, for LC-MS analysis, the R^2^Y value was 0.969, and the Q^2^ value was 0.886 (Table S1). Notably, across both analytical platforms, the R^2^ and Q^2^ values on the left of the plots were consistently smaller than the initial values on the right, further confirming the model’s stability (Figure 1E,F). Taken together, these findings indicate that the metabolism of *B. gargarizans* undergoes dramatic changes during hibernation.

### 3.2. Differential Metabolite Profiles Between Hibernation and Non-Hibernation Toads

**Figure 2:**
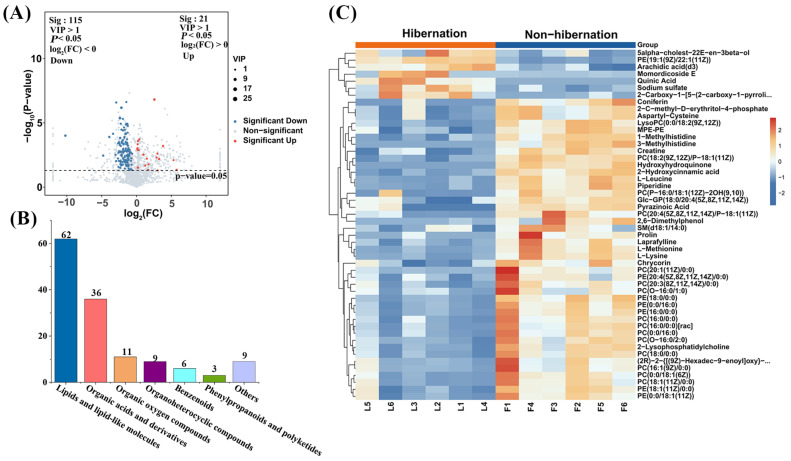
(**A**) Volcano plot of the differentially expressed metabolites (DEMs) between hibernation and non-hibernation groups. Red and blue dots indicate significantly upregulated and downregulated metabolites, respectively. (**B**) Distribution of 136 DEMs categorized by class. (**C**) Heat map showing the concentration levels of DEMs in the hibernation and non-hibernation groups. Red indicates higher concentrations, while blue indicates lower concentrations. Metabolites were considered differential with VIP > 1 and *p* < 0.05 (*n* = 12).

A total of 136 differentially expressed metabolites (DEMs) were identified between the hibernation and non-hibernation groups, based on criteria of VIP > 1 and *p* < 0.05. Among these, 21 metabolites were found to be upregulated, while 115 metabolites were downregulated in hibernating toads compared to non-hibernating individuals (Figure 2A, Table S2). The DEMs included 62 lipids and lipid-like molecules, 36 organic acids and derivatives, 11 organic oxygen compounds, 9 organoheterocyclic compounds, 6 benzenoids, 3 phenylpropanoids and polyketides, and 9 other compounds (Figure 2B).

The predominance of lipids and organic acids implies substantial shifts in amino acid, carbon, and lipid metabolism during hibernation. Of these lipids, 35 were identified as phosphatidylcholines (PCs) or phosphatidylethanolamines (PEs), which are essential for maintaining cellular membrane integrity and facilitating signal transduction. To clarify the relationships between samples and the top 50 DEMs, a cluster analysis was conducted across 12 biological replicates (Figure 2C). The heatmap indicates that only seven DEMs, such as sodium sulfate, quinic acid, momordicoside E, and arachidic acid (d3), were upregulated in the hibernation group. Conversely, 43 DEMs, including L-Leucine, 2-Lysophosphatidylcholine, 2-Hydroxycinnamic acid, and 1-Methylhistidine, were significantly more abundant in the non-hibernation group. These distinct metabolite profiles reflect significant metabolic shifts between the hibernation and non-hibernation periods.

### 3.3. KEGG Analysis of Metabolic Pathway Differences

**Figure 3:**
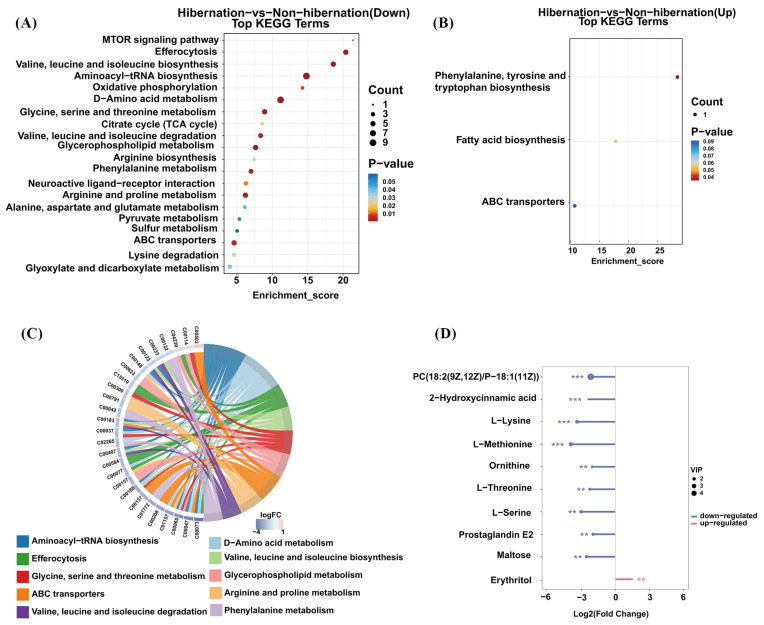
(**A**,**B**) Bubble maps of downregulated and upregulated KEGG pathways for DEMs between hibernation and non-hibernation groups (*p* < 0.05). The vertical ordinate shows metabolic pathways, while the horizontal ordinate indicates the rich factor. Larger bubbles signify more DEMs associated with a pathway, and bubble color transitions from blue to red with decreasing *p*-values. (**C**) Chord diagrams show the top 10 pathways from KEGG enrichment analysis, selected based on *p*-value < 0.05 and listHits > 1. Red indicates upregulation, while blue indicates downregulation; DEMs are on the left, with selected pathways on the right. (**D**) The lollipop map illustrates DEMs enriched in the top 10 pathways, with red representing upregulation and blue representing downregulation. Significant differences are marked by ** (*p* < 0.01) and *** (*p* < 0.001).

To investigate the changes in metabolic pathways between the two groups, we annotated 136 DEMs to 52 metabolic pathways using the KEGG database. Analysis revealed that most pathways in the hibernation group were downregulated (Figure 3A). These include pathways such as pyruvate metabolism; efferocytosis, valine, leucine, and isoleucine biosynthesis; the citrate cycle (TCA cycle); glycerophospholipid metabolism; and arginine and proline metabolism. In contrast, only three pathways were upregulated in the hibernation group: phenylalanine, tyrosine, and tryptophan biosynthesis, fatty acid biosynthesis, and ABC transporters (Figure 3B).

To further examine the relationship between DEMs and metabolic pathways, we focused on the top 10 pathways that showed significant differences (*p*-value < 0.05) and included more than one differential metabolite (listHits > 1). As shown in Figure 3C,D, the analysis indicated that among the metabolites associated with these pathways, all except erythritol (e.g., L-lysine, L-serine, L-methionine, ornithine) were significantly downregulated in the hibernation group. These results underscore the broad suppression of essential metabolic pathways during hibernation, including amino acid metabolism, lipid metabolism, carbon-derived energy production, and cellular signaling processes. This suppression underpins the physiological adaptations essential for survival, aiding in energy conservation and resource efficiency.

### 3.4. Metabolic Network

**Figure 4:**
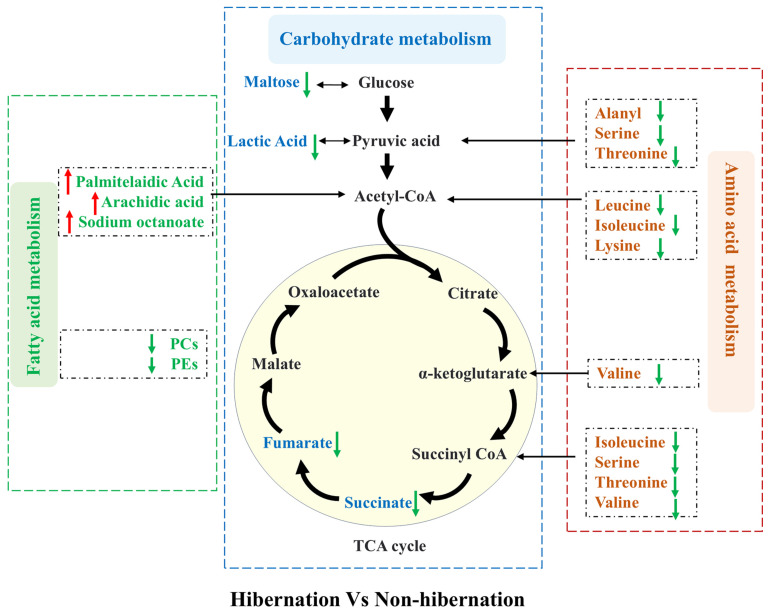
Relative differences in metabolites associated with amino acid, carbohydrate, and lipid metabolism in the hibernation group compared to the non-hibernation group. Green arrows indicate metabolites that are downregulated in the hibernation group relative to the non-hibernation group, while red arrows indicate upregulated metabolites. The schematic highlights changes in carbohydrate metabolism (blue box), amino acid metabolism (brown box), and fatty acid metabolism (green box), along with alterations in the tricarboxylic acid (TCA) cycle.

A comprehensive metabolic network map was constructed based on the roles of DEMs (Figure 4). In amino acid metabolism, DEMs such as L-Leucine, L-Valine, L-Serine, and L-Proline are converted into metabolic intermediates like pyruvate, alpha-ketoglutaric acid, acetyl CoA, and succinyl CoA through processes such as deamination, acylation, and decarboxylation, ultimately providing energy for physiological functions. Notably, these amino acids are significantly reduced in the hibernation group. Conversely, lipid metabolism in the hibernation group shows a significant upregulation of fatty acids involved in the β-oxidation process, including palmitelaidic acid, arachidic acid, and sodium octanoate, indicating that lipids may serve as a primary energy source for toads during hibernation. DEMs involved in carbon metabolism, such as fumarate, succinate, maltose, and lactic acid, are markedly decreased in the hibernation group, suggesting a decreased energy requirement during this period. Overall, the findings demonstrate a distinct metabolic shift in *B. gargarizans* during hibernation, characterized by a reduction in amino acid and carbon metabolism, alongside an upregulation of lipid metabolism.

## 4. Discussion

In this study, we investigated the metabolic changes in *B. gargarizans* during hibernation using GC-MS and LC-MS metabolomics approaches. We identified significant shifts in metabolic profiles between hibernation and non-hibernation groups, particularly in the pathways of amino acids, lipids, and carbohydrates. The reduction in amino acid metabolism suggests an energy conservation strategy characterized by decreased protein synthesis and degradation. Concurrently, enhanced β-oxidation of fatty acids underscores the critical role of lipids as a primary energy source. Additionally, a reduction in the levels of most PCs and PEs may contribute to maintaining membrane functionality and stability under low-temperature conditions. Collectively, these changes highlight *B. gargarizans*’ optimized energy use and unveil adaptive strategies critical for survival in cold environments, demonstrating essential physiological adaptations for hibernation.

During hibernation, animals adapt to harsh environmental conditions by minimizing their energy expenditure [25,26]. In our study, 115 out of 136 DEMs, including those involved in energy metabolism such as amino acids, lipids, and lactic acid, were downregulated in hibernating individuals compared to active toads, demonstrating a suppression of metabolic activity. This reduction likely reflects an adjustment of the energy supply and demand balance, and specifically a decrease in energy demand during hibernation. Reduced levels of amino acids suggest a slowing of protein synthesis and amino acid conversion processes, which may help conserve amino acid reserves in the absence of dietary intake [19,27]. Notably, of the 136 DEMs, 62 were lipids and lipid-like molecules. Among these, PCs and PEs were predominant and significantly downregulated. PCs and PEs are crucial components of phospholipids involved in cell membrane formation and lipid signaling. The decrease in metabolic rate and physiological activity during hibernation likely leads to reduced synthesis and the renewal of cell membranes, as well as diminished lipid signaling, resulting in lower PC and PE levels [28]. Disruption in the PC/PE ratio has also been shown to be associated with hepatocyte dysfunction and metabolic dysregulation in both mice and humans, highlighting the critical role of these phospholipids in maintaining cellular and metabolic homeostasis [29,30]. In contrast, we observed a significant upregulation of certain fatty acids involved in β-oxidation, such as palmitic acid, arachidonic acid, and sodium caprylate, highlighting the role of lipids as a major energy source during dormancy [31,32]. These alterations in the overall metabolic profile of *B. gargarizans* during hibernation align with the decreases in energy consumption requirements.

KEGG pathway analysis revealed extensive metabolic remodeling in hibernating *B. gargarizans*, characterized by widespread metabolic suppression. Specifically, pathways associated with energy metabolism, including pyruvate metabolism, the citrate cycle (TCA cycle), and oxidative phosphorylation, were significantly downregulated, suggesting a strategic metabolic adaptation to minimize ATP production and reduce expenditure during hibernation [33]. Studies have shown that under energy-limited conditions, energy sensors such as AMP-activated protein kinase (AMPK) regulate metabolic processes to suppress ATP-consuming pathways and promote energy-efficient mechanisms [34]. Additionally, mitochondrial modifications, such as the downregulation of electron transport chain activity, have been suggested as critical adjustments to align energy supply and demand during hypometabolic states [35]. Similarly, amino acid metabolic pathways, such as glycine, serine, and threonine metabolism, D-amino acid metabolism, and lysine degradation, also showed significant downregulation, indicating reduced protein synthesis and amino acid interconversion. The downregulation of aminoacyl-tRNA biosynthesis indicated the suppression of protein translation processes, underscoring a reduction in cellular growth and repair activities during hibernation [19]. Glycerophospholipid metabolism and the degradation pathway of branched-chain amino acids, including valine, leucine, and isoleucine, were similarly downregulated. This inhibition likely reflects a reduction in cell membrane renewal and lipid signaling, as glycerophospholipids and branched-chain amino acids play essential roles in maintaining cell membrane homeostasis [36,37,38]. Hibernating amphibians, such as *Nanorana parkeri* and *Strauchbufo raddei*, demonstrate consistent patterns of metabolic suppression, particularly in lipid and amino acid metabolism. This adaptive metabolic reprogramming appears to be a conserved strategy that enables energy conservation, enhances stress tolerance, and maintains cellular stability during extended periods of dormancy under harsh environmental conditions [19,39]. Moreover, signaling pathways such as the mTOR signaling pathway, neuroactive ligand–receptor interactions, and efferocytosis were also downregulated, indicating the suppression of cellular growth, immune regulation, and neural activity during hibernation [40,41]. Despite the widespread downregulation of metabolic pathways, a subset of pathways, including fatty acid biosynthesis and phenylalanine, tyrosine, and tryptophan biosynthesis, remained active. These pathways likely support the essential functional requirements of cell membranes or provide lipids and aromatic amino acids as critical energy and signaling molecules during hibernation [42,43].

The constructed metabolic network highlights key metabolites across amino acid, lipid, and carbohydrate metabolisms, elucidating the metabolic adaptations in hibernating *B. gargarizans*. Amino acids such as L-serine, L-leucine, L-valine, and L-isoleucine undergo transformations through deamination, acylation, and decarboxylation into intermediate metabolites like pyruvate, acetyl-CoA, α-ketoglutarate, and succinyl-CoA. These intermediates facilitate the TCA cycle, thereby supporting essential physiological functions [44]. However, there is a notable reduction in amino acid metabolism within hibernating toads. In contrast, the upregulation of fatty acids involved in β-oxidation, such as palmitelaidic acid, arachidic acid, and sodium octanoate, indicates an increased reliance on lipid metabolism during hibernation. This shift is beneficial for minimizing protein catabolism and thus preserving muscle tissue under prolonged low-temperature conditions [45]. Additionally, the observed reduction in key carbohydrate metabolism intermediates, such as fumaric acid, succinic acid, maltose, and lactic acid, supports the hypothesis of reduced energy requirements in hibernating *B. gargarizans*.

## 5. Conclusions

Our study offers a comprehensive understanding of the metabolic adaptation strategies of *B. gargarizans* during hibernation. As expected, metabolites and pathways related to amino acid and carbohydrate metabolism demonstrated a significant suppression, consistent with an overall reduction in metabolic activity. This is evidenced by the decreased levels of key TCA cycle intermediates, such as fumaric acid and succinic acid, which directly reflect lower energy production demands during hibernation. In contrast, lipid metabolism exhibited more complex and specific changes. Notably, while the levels of many fatty acids, such as PEs and PCs, decreased, certain fatty acids associated with β-oxidation, including palmitoleic acid, arachidonic acid, and sodium caprylate, showed an increase. These findings highlight the critical role of lipid β-oxidation as a primary energy source during hibernation. This metabolic remodeling reflects a strategic allocation of energy resources to withstand the challenges posed by cold and nutrient scarcity. Our findings provide novel insights into the metabolic adaptation mechanisms of amphibian hibernation and establish a foundation for future research aimed at uncovering the underlying molecular regulatory pathways involved.

## Data Availability

The metabolic data analyzed are presented in this article. The raw data are available from the Genome Sequence Archive (GSA) under the accession number OMIX008514.

## Supplementary Materials

The following supporting information can be downloaded at www.mdpi.com/article/10.3390/ani15030403/s1, Table S1: Permutation test of the OPLS-DA model.xlsx; Table S2: Differential metabolites identified between the hibernation and non-hibernation groups.xlsx.

## Abbreviations

The following abbreviations are used in this manuscript:

DEMs	Differentially expressed metabolites;
PEs	Phosphatidylethanolamines;
PCs	Phosphatidylcholines;
LC-MS	Liquid chromatography–mass spectrometry;
GC-MS	Gas chromatography–mass spectrometry;
PCA	Principal component analysis;
OPLS-DA	Orthogonal partial least squares discriminant analysis;
KEGG	Kyoto encyclopedia of genes and genomes.
